# Effect of hip abductor strengthening exercises in knee osteoarthritis: a randomized controlled trial

**DOI:** 10.1186/s12891-020-03316-z

**Published:** 2020-05-07

**Authors:** Varah Yuenyongviwat, Siwakorn Duangmanee, Khanin Iamthanaporn, Pakjai Tuntarattanapong, Theerawit Hongnaparak

**Affiliations:** Department of Orthopedics, Faculty of Medicine, Prince of Songkla University, Hat Yai, Songkhla, 90110 Thailand

**Keywords:** OA knee, Hip abductor exercise, KOOS

## Abstract

**Background:**

Osteoarthritis knee (OA) for patients whom had varus malalignment had higher peak adductor moment. Hip abductor strength played an important role in the decreasement of knee adduction moment. This study aimed to evaluate the effect of hip abductor exercises for patients who had medial compartment knees OA.

**Methods:**

Patients who had medial compartmental OA knee were randomized into two groups. The first group performed hip abductor strengthening exercises, combined with quadriceps strengthening exercises; whereas, the second group performed standalone quadriceps strengthening exercises. Self-reported Knee Injury and Osteoarthritis Outcome Scores (KOOS) were collected by patients on follow-up visits.

**Results:**

Eighty-six patients completed the trial. All KOOS subscales were significantly improved in both groups after 10 weeks of treatment. However, there was no significant difference in the scores between either group at 2–10 weeks after treatment. Nevertheless, the effects of exercise for pain, symptoms, function in daily living and knee-related quality of life were found to have faster improvement within the hip abduction exercise group compared to the control group (2 weeks faster; pain, function in daily living and knee-related quality of life, 4 weeks faster; symptoms.)

**Conclusion:**

Since, adding quadriceps exercises could expedite improvement of less pain, symptoms, activity in daily living and quality of life faster than quadriceps exercises solely for a 2–4 weeks period. However, the effect size was small and there were no differences after this; hence, consideration of adding hip abductor exercises in the treatment protocol should be based on the patients and doctors appraisal.

**Trial registration:**

TCTR, TCTR20180517005. Registered 17 May 2018.

## Background

Knee osteoarthritis (OA) is a disease that produces pain and limits the functional movement of patients. Whilst there are many clinical practice guidelines for treatment of this condition [1–3], all clinical practice guidelines emphasize non-pharmacological approaches; particularly exercise therapy [1–3]. Although, there were many exercises that are purposed for treatment of knee osteoarthritis [4] Quadriceps strengthening exercises have been a cornerstone for treatment for OA knee patients [5], as it has the benefit of reducing patient symptoms and preserves function [6]. Another exercise, that is a specific muscle group exercise, is hip abductor exercises [7]. There have been a number of studies reporting the effectiveness of hip abductor exercises for treatment of medial tibiofemoral OA, in which they reported the benefits of hip abductor strength for improving pain, physical function, muscle strength [5] and reduced risks of 2-year tibiofemoral cartilage damage, which was evaluated by magnetic resonance imaging (MRI) [8].

OA knee patients, especially for patients who have varus malalignment, had higher peak adduction moments in comparison to the normal population [9, 10]. Knee adduction moment is the moment that acts on the joint in the frontal plane. This acts to rotate the tibia medially on the femur while walking [11]. Knee adduction moment tends to force the knee outwards, compressing the medial joint compartment and stretching the lateral joint structures [12]. This adduction moment also relates to the severity and progression of the disease, higher adduction moments are related with a higher rate of disease progression [13, 14]. High, peak knee adduction moments were positively associated with greater pain, and were negatively associated with the knee functional score in OA knees [15].

There was a report stating that hip abductor muscle strength played a major role for reducing knee adduction moments, because it counteracts pelvic drop in the contralateral swing limb during the single-limb stance phase of gait. This intensifies forces at the medial compartment knee of the stance limb [16, 17].

Theoretically, the higher hip abduction strength, the lower knee adduction moment, which could lower pain and improve function in OA knee patients.

Previous studies that compared hip abductor strengthening exercises in OA knee patients, against control patients who did not undergo any strengthening exercise programs revealed that: the exercise group had lesser knee pain and better performance-based physical function than that of the control group [5]. There was also a small randomized study that reported improved Western Ontario and McMaster Universities Arthritis Index (WOMAC) and the 6 min walk test in hip abductor strengthening exercise [18]. However, this was a limit study that evaluated the benefits of hip abductor strengthening exercises combined with quadriceps exercises, which was the most widely used recommendation, comparing with quadriceps strengthening alone in terms of pain and other aspects. Our study aimed to evaluate the effect of hip abductor exercises combined with quadriceps exercises in patients who had OA knee. The authors considered performing the study in medial compartment OA knees only, this was based on the theory that abnormal lower limb adductor moment might have more effect at the medial compartment of the knee [9, 10].

## Method

This study was a prospective randomized control trial. The study was conducted at a tertiary care hospital, from; May, 2018 to December, 2019. This study was approved by the local ethics committee and institutional review board. The procedures in this study were performed under the Declaration of Helsinki’s ethical principles for medical research involving human participants. Written informed consent was obtained from all individual participants included in the study.

### Participants

Patients who had medial compartmental knee osteoarthritis, were aged 50 years and above, able to walk without gait aids, could flex the knee more than 90 degrees, had knee alignment with a varus of less than 10 degrees as well as plain standing radiographs; showing medial compartment OA with Kellgren and Lawrence classification (KL-classification) II-III in knee radiographic, were included into the study. The exclusion criteria’s were patients who had inflammatory arthritis, osteoarthritis of the hips, having had previous knee or hip surgery, received intraarticular injection within 6 months, and patients who had neurological and muscle problems.

### Accounting for all patients

**Fig. 1:**
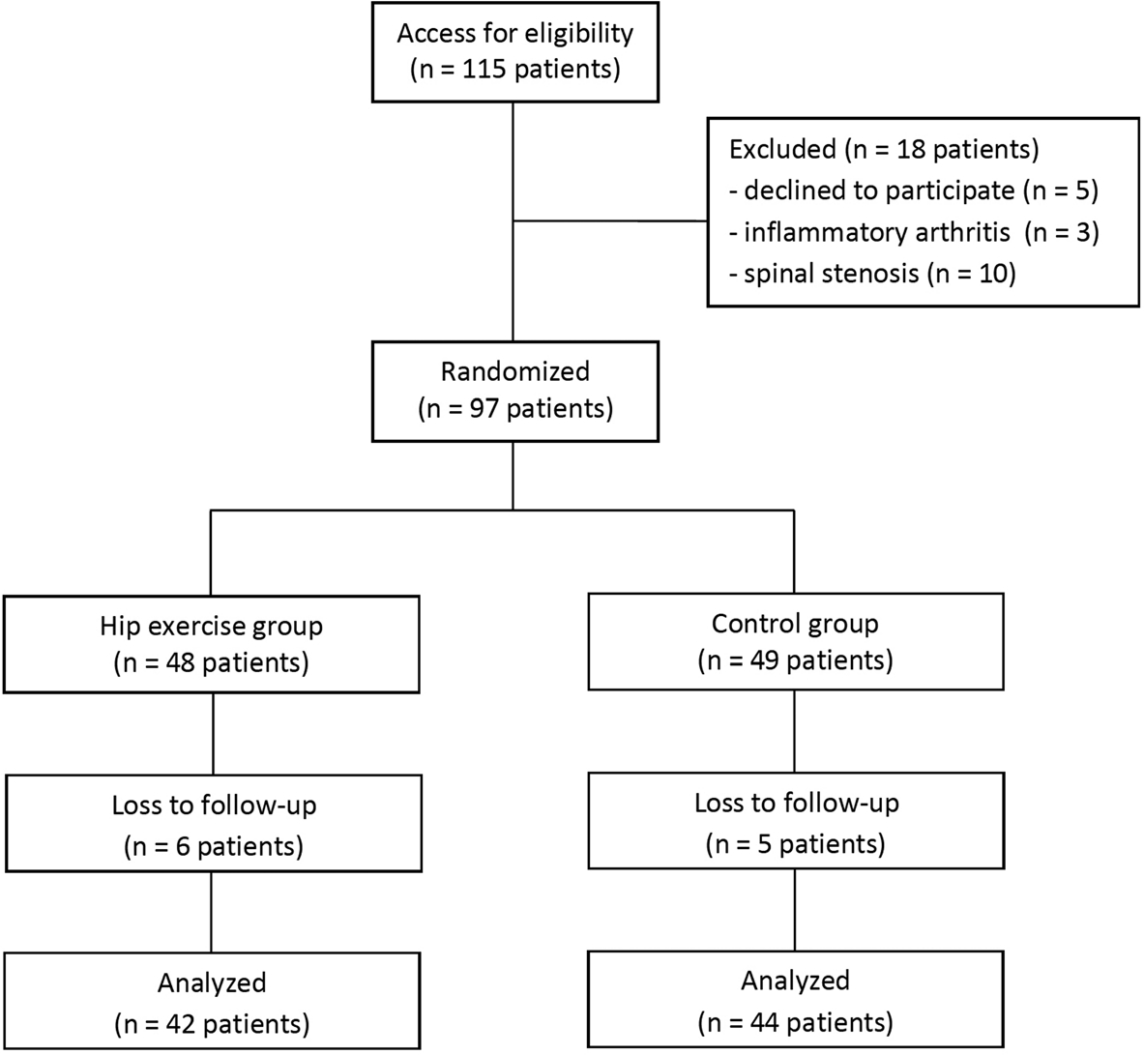
A diagram of the study enrollment process

One hundred and 15 patients were approached for the study, 5 patients declined to participate, 3 patients had inflammatory arthritis and 10 patients had spinal stenosis. Finally, 97 patients participated in the study, from these 86 patients completed the trial and were subsequently analyzed. Eight patients (4 patients in each group) considered withdrawing from the study by themselves after first visit, because they felt improvement of symptoms. Two patients in the hip abductor exercise group along with one patient in the control group did not attend their 2-week follow up appointments, and they could not be contacted by the research assistance. Intention-to-treat analysis was performed in this study. (Fig. 1).

### Randomization

Random numbers were generated by computer, upon which the Block-of-four randomization method was used for randomizing patients into two groups. Sealed, opaque envelopes were used for allocating patients, and were only opened in the rehabilitation unit after patients were enrolled in the study.

The first group of patients performed hip abductor strengthening exercises combined with quadriceps strengthening exercises. Patients received instructions on how to perform these exercises by a physical therapist. The patients performed quadriceps exercises and hip abductor exercises following the protocol: 3 days/ weeks (Table 1). Knee quadriceps exercises were performed by patients sitting in a chair and flexing their knee to a 90-degree flexion position, then gradually extending the knee to full extension, holding this position for 10 s; before flexing the knee. The hip abductor exercise was done by patients lying down in a side-lying position and abducting the hip to a 45-degree abduction position, then holding this position for 10 s. While performing either quadriceps exercises or hip abduction exercises, the ankle of the patient was strapped with a sandbag, which as weighted as per protocol. Both knee and hip exercises were performed as a combination of 4 sets- of 10 repetitions in the morning, and then again in the evening; for 3 days a week. The second group was the control group, patients performed only quadriceps strengthening exercises with the same protocol. All patients were prescribed naproxen as rescue medication, which would be occasionally used upon patient demand, but no more than twice daily in case of moderate-severe pain, or pain limit patient function. In case of being allergic to naproxen, tramadol was used instead.

**Table 1:** Exercise protocol

Week	Weight (Repetitive maximum; RM)
1–2	50% of 10 RM
2–4	60% of 10 RM
4–6	70% of 10 RM
6–8	80% of 10 RM
8–10	90% of 10 RM

Both groups of patients were taught with the same patient education program for self-care, such as activity modification, sport and weight control. Patients were followed up at 2 weeks, 4 weeks, 6 weeks, 8 weeks and 10 weeks.

Self-reported Knee Injury and Osteoarthritis Outcome Scores (KOOS) were collected by patients on each follow-up visit. The score consisted of 5 separately scored subscales; Pain, Symptoms, Function in daily living (ADL), Function in Sport and Recreation (Sport/Rec) and knee-related Quality of Life (QOL). The score is from 0 to 100, 0 representing extreme problems and 100 representing no problems [19]. The amount of analgesic used for rescue medication was recorded by patients.

### Statistical analysis

Analyses were performed using R version 3.1.0 software (R Foundation for Statistical Computing, Vienna, Austria). Patient demographic data, such as age, weight, height, Body Mass Index (BMI) and tibiofemoral angle were evaluated with t-test. Gender, side of osteoarthritis, history of diabetes, KL-classification and rescue medication usage were compared with Fisher’s exact test. KOOS were analyses via t-test in every subscale. Generalized estimating equation (GEE) modelling was used for studying the longitudinal association between type of exercise, and KOOS in each subscale. We considered the minimum clinically important differences (MCID) of KOOS on the 100-point scale to be 8 points, based on prior evidence [19].

## Results

Baseline demographic data were not different between groups in terms of age, gender, weight, height, BMI, side, KL-classification and history of diabetes (Table 2).

**Table 2:** Demographic data

Characteristic	Hip exercise group *n* = 42	Control group *n* = 44	*p* value
Age (years)	62.8 ± 6.80*	62.5 ± 8.4*	0.85
Sex (male:female)	4:38	3:41	0.71
Weight (kg)	65.3 ± 11.7*	61.2 ± 9.1*	0.07
Height (cm)	158.1 ± 7.4*	156.5 ± 6.3*	0.26
BMI (kg/m^2^)	26.2 ± 5.2*	25.1 ± 4.4*	0.3
Side (right:left)	20:22	21:23	1.0
KL-classification (II/III)	16:26	16:28	0.87
Tibiofemoral angle	180.86 ± 2.96*	180.71 ± 2.57*	0.65
Diabetes	2	3	1.0

* Mean values with SDs

Knee injury and Osteoarthritis Outcome Scores were not significantly different between both groups of patients at the start of treatment. (Pain; *P* = 0.39, Symptom; *P* = 0.57, ADL; *P* = 0.27, Sport/Rec; *P* = 0.66, QOL = 0.62) All subscales were also not significantly different between both groups at 2 weeks, 4 weeks, 6 weeks, 8 weeks and 10 weeks after treatment (*P* > 0.05). GEE analyses showed both groups also had no difference in each subscale at 10 weeks. (Table 3).

**Table 3:** Mean Knee Injury and Osteoarthritis Outcome Scores (KOOS)

		Baseline	2 wk	4 wk	6 wk	8 wk	10 wk	*p* value
Pain	Hip exercise	70	79	85	86	88	89	0.67
	Control	74	79	82	84	88	91	
Symptoms	Hip exercise	76	86	86	84	87	90	0.22
	Control	80	84	85	86	91	89	
Activities of daily living	Hip exercise	77	84	91	91	94	92	0.77
	Control	81	86	88	90	92	95	
Sports and recreation	Hip exercise	29	34	32	39	40	47	0.57
	Control	32	37	38	42	45	55	
Quality of life	Hip exercise	48	57	62	66	86	72	0.61
	Control	50	58	61	64	70	77	

**Fig. 2:**
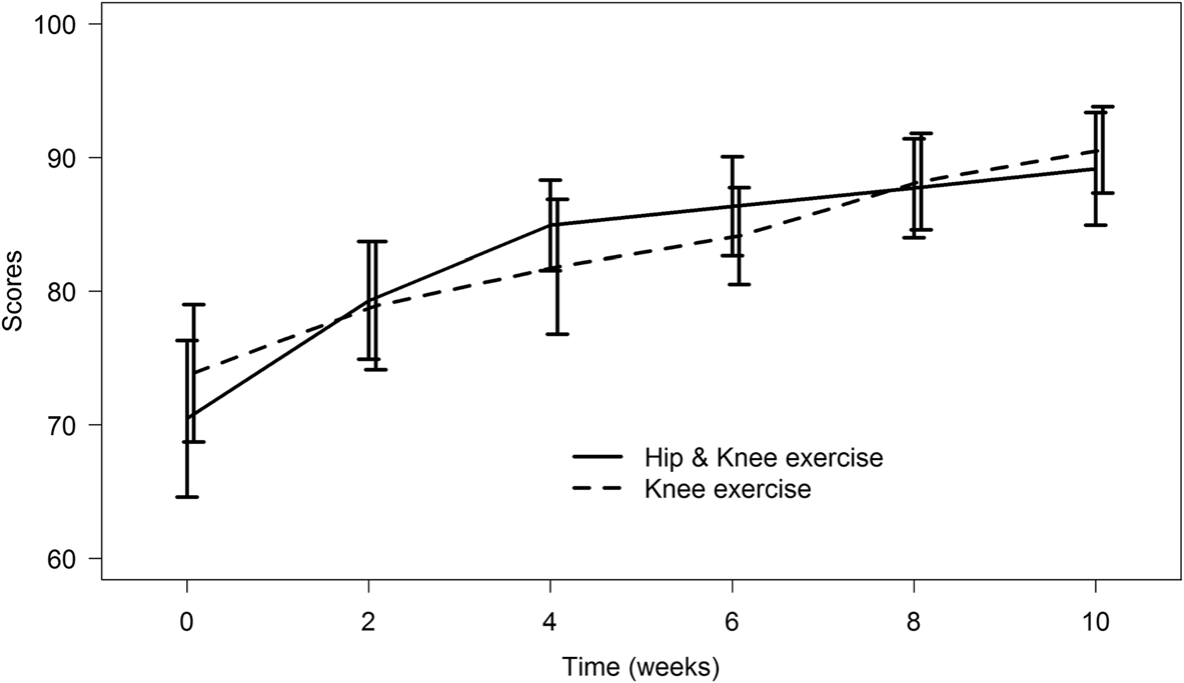
Knee Injury and Osteoarthritis Outcome Scores (KOOS) subscale; Pain

GEE analyses revealed both groups had significantly improved KOOS pain at 10 weeks. (Hip abductor exercise group + 18.68 (95% CI, 11.8–25.6, *P* = < 0.01), Control group; + 16.69 (95% CI, 10.9–22.5, *P* < 0.01). (Fig. 2) The other subscales also showed improvement at 10 weeks (P < 0.01) (Table 4).

**Table 4:** Mean different from base line (95% CI) of Knee Injury and Osteoarthritis Outcome Scores (KOOS)

		2 wk	4 wk	6 wk	8 wk	10 wk
Pain	Hip exercise	8.83 (1.82–15.8) *p* = 0.01	14.45 (7.97–20.9) *p* < 0.01	15.88 (9.25–22.5) *p* < 0.01	17.23 (10.6–23.8) *p* < 0.01	18.68 (11.8–25.6) *p* < 0.01
	Control	5.05(−1.69–11.8) *p* = 0.14	7.94 (1.04–14.8) *p* = 0.02	10.24 (4.22–16.3) *p* < 0.01	14.32 (8.30–20.3) *p* < 0.01	16.69 (10.9–22.5) *p* < 0.01
Symptoms	Hip exercise	9.95 (3.14–16.8) *p* < 0.01	9.85 (2.97–16.7) *p* < 0.01	8.23 (1.39–15.1) *p* < 0.01	11.75 (4.83–18.7) *p* = 0.02	14.65 (8.20–21.1) *p* < 0.01
	Control	3.68(−2.70–10.1) *p* = 0.26	5.14 (− 1.14–11.4) *p* = 0.11	6.21 (0.38–12) *p* = 0.04	10.47 (4.70–16.3) *p* < 0.01	9.09 (2.84–15.3) *p* < 0.01
Activities of daily living	Hip exercise	8.14 (1.05–15.2) *p* = 0.02	14.05 (7.78–20.3) *p* < 0.01	14.84 (8.63–21) *p* < 0.01	17.08 (11.1–23.0) *p* < 0.01	15.19 (8.77–21.6) *p* < 0.01
	Control	4.91(−1.86–11.7) *p* = 0.16	7.29 (0.38–14.2) *p* = 0.04	8.72 (1.98–15.5) *p* = 0.01	11.18 (4.47–17.9) *p* < 0.01	13.84 (7.29–20.4) *p* < 0.01
Sports and recreation	Hip exercise	5.24(−5.54–16) *p* = 0.34	3.89(−6.02–13.8) *p* = 0.44	9.99(− 1.14–21.1) *p* = 0.08	11.22(− 1.35–23.8) *p* = 0.08	17.79 (5.08–30.5) *p* < 0.01
	Control	4.55(− 13.8–7.53) *p* = 0.4	6.25(− 6–15.1) *p* = 0.24	10.13(− 4.22–16.7) *p* = 0.07	13.34(− 0.74–21) *p* = 0.02	22.69 (2.6–24.1) *p* < 0.01
Quality of life	Hip exercise	8.4 (0.35–16.5) *p* = 0.04	13.92 (5.38–22.5) *p* < 0.01	17.28 (8.59–26) *p* < 0.01	19.92 (11–28.9) *p* < 0.01	24.12 (15.6–32.6) *p* < 0.01
	Control	7.32(− 0.36–15) *p* = 0.06	10.2 (2.33–18.1) *p* = 0.01	13.74 (6.09–21.4) *p* < 0.01	20.1 (12.4–27.8) *p* < 0.01	27.09 (19.3–34.9) *p* < 0.01

The effects of exercise for pain management along with many subscales were found to be faster in the hip abduction exercise group, compared to the knee exercise only group. The hip abductor exercise group had significant improvement of lesser pain, improved QOL and ADL after 2 weeks, while the control group had significantly better results at 4 weeks. Symptoms improved in the hip abductor exercise group at 2 weeks; while the control group showed improvement at 6 weeks. (Table 4) However, function in Sport and Recreation improved faster in the control group, which improved at 8 weeks; whereas, the hip abductor exercise group improved at 10 weeks.

All patients used naproxen as rescue medication, without allergic reactions. There was no difference in terms of rate of rescue medication usage between both groups, at any time point. (0–2 wk. [hip exercise group 0%, control group 9.1%,*P* = 0.18], 2-4wk [hip exercise group 4.8% / control group 9.1%, *P* = 0.68], 4–6 wk. [hip exercise group 9.5% / control group 11.4%, *P* = 1], 6-8wk [hip exercise group 2.4% / control group 2.3%, *P* = 1], 8–10 wk. [hip group 4.8% / control group 4.5%, *P* = 1]).

## Discussion

Strengthening exercises are a general accepted treatment for OA knees. Quadriceps exercises were studied, and reported the effectiveness for reduced pain and improved function in OA knees [20, 21]. Hip abductor exercises were of interest as an adjuvant exercise for OA knee patients [7]. This was based on the hypothesis that gluteus medius muscles effected external knee adduction moment during level walking [22]. In so saying, the authors conducted this study to evaluate the efficacy of hip abductor exercises in combination with quadriceps exercises, as a comparison to quadriceps strengthening alone, for patients who had medial compartment knee OA. Our study found that adjuvant hip abductor exercises coupled with quadriceps exercises helped improve pain reduction and function in OA knee patients to the same degree as patients who performed quadriceps exercise alone, but added hip abductor exercises did have a faster effect in the reduction of both pain and function.

This study had a number of limitations. First, patients were not blinded to the exercise protocol, because the strengthening exercises required patient participation. So, patients might have had biases, in that the exercise protocol might have had an effect on both symptoms and function. Second, our study had a high proportion of females. This being said, based on theory, we believe that our results should be able to be applied to male patients equally. Third, this study reported the results in only mild and moderate severity of OA knee patients. So, further studies in more severe cases would be of interest. Finally, we did not exclude patients who might have self-home exercise therapy or any aerobic exercise prior to participation in the study, which might have influenced the results. However, no patients had ever undergone our education program, or rehabilitation treatment protocol prior to this study.

This study found that hip abductor exercises combined with quadriceps exercises, or quadriceps exercises alone could improve patient pain and function in medial compartment OA knees. Previous studies on hip abductor exercises had the same results. Bennell KL et al. compared the effects in a hip abductor exercise group against a control group who performed no exercises, and this report stated that: the hip exercise group had significantly greater improvement in pain reduction and physical function [5]. There was also a small randomized study, containing 30 patients, which compared a hip abductor strengthening group combined with quadriceps, along with a control group that performed only quadriceps exercises. From this study the results showed both groups had improvement of WOMAC scores after treatment [18].

Our results demonstrated that our hip abductor exercise group had no difference in the improvement of pain and function when compared with those only doing quadriceps exercises; from a longitudinal prospective. Our study did however have contradicting results with this previous, small randomized trial that reported a better WOMAC physical function scale, and 6 min walk test (6MWT) in a hip abduction exercise group, which combined quadriceps exercises in comparison to standalone quadriceps exercises after 6 weeks of treatment [18].

However, we found that our hip abductor exercise group had earlier improvement than that of the quadriceps exercise group for pain, symptom, activity in daily living and quality of life. Our study considered 8 points for the minimum clinically important differences of KOOS, based on prior evidence [19]. The mean difference of KOOS at 2 weeks, and baseline in the hip exercise group, which had statistically significant improvement, were also above the minimum clinical importance range (range 8.14–9.95). However, the effect size of these results; even though greater than the minimum clinically important difference, was small. Moreover, the difference only appeared to be over a 2–4 weeks period, after which both groups obtained the same level at the end of the treatment.

To our knowledge, our study is the first study that both controlled and evaluated rescue medication usage, in which we found that there was no difference between either group. Further studies in patients with more severity, or a study combined with gait analysis to evaluate knee adduction moment differences should be expanded, so as to evaluate the benefits of this exercise.

## Conclusion

In conclusion, we found that either hip abductor exercises combined with quadriceps exercises or quadriceps exercises alone could lessen patient pain and improve function. Adding quadriceps exercises could expedite improvement of less pain, symptoms, activity in daily living and quality of life faster than quadriceps exercises alone; however, this only appeared to be over a 2–4 weeks period with small effect size, after which there was there were no differences. Hence, considering to add hip abductor exercises in the treatment protocol should be based on the patients and doctors perspective.

## Data Availability

The datasets generated during this current study are available from the corresponding author upon reasonable request.

## Abbreviations

OA: Osteoarthritis
WOMAC: Western Ontario and McMaster Universities Arthritis Index
KOOS: Knee Injury and Osteoarthritis Outcome Scores
ADL: Function in daily living
Sport/Rec: Function in Sport and Recreation
QOL: Knee-related Quality of Life
KL-classification: Kellgren and Lawrence classification
